# Annotations

**Published:** 1895-03-09

**Authors:** 


### ANNOTATIONS.

### Influenza Convalescents.

If all the men and women who are now convalescing from influenza in London alone were placed side by side in regiments they would constitute a very considerable army. To tell all these in a single comprehensive word what they ought to do to get speedily and thoroughly well would be a worthy public service. A leading article in one of the evening papers the other day, intending only to utter a pleasant jest, gave its readers this little bit of Latin rhetoric, which, though we have not verified it, has a smack of the Prayer Book: "*Influenza afflavit, et dissipati sunt.*" The influenza has blown upon them, and they are scattered. That our contemporary had no intention of poaching upon medical preserves, and recommending a line of treatment for influenza convalescents, we are quite sure. Nevertheless, *dissipati sunt* is full of important therapeutic suggestion. If influenza convalescents are not "scattered," they ought to be. That is the secret of quick recovery. The man or woman who has had a mild attack of the malady should go to some mild, but dry and bracing, seaside or inland health resort for a few days before resuming active work. Those who have had more severe attacks should go for a few weeks. This, in nine cases out of ten, will be found to be a real saving of time, money, health, and temper for the busy man. It is almost impossible to effectually throw off an attack of influenza whilst remaining in London. The rule, always and everywhere, if possible, should be this: *Influenza afflavit, et dissipati sunt.*

### Football Players' Impetigo (?)

A mysterious skin affection having been reported as prevalent amongst school boys who play football, the Council of the Medical Officers of Schools Association have instituted an inquiry as to the nature of the disease. Information as to the circumstances of its origin, its features, its contagiousness, and its treatment is desired, and may be sent to Dr. Armstrong, of Wellington College, Berks. An opinion has been expressed that the affection is impetigo contagiosa, characterised by the presence of the staphylococcus pyogenes aureus, and that its special prevalence in football players is due to the susceptibility of skins abraded in scrimmages to the infective germs of the disease. The disease is said to occur principally on the face, ears, and neck, and sometimes on the hairy scalp; and its stages are those of erythema, papule, vesicle, and purulent crust, the latter when on the head having the appearance of porrigo. We notice that in the schedule no inquiry is made as to the condition of the jerseys worn by affected players, which we think an important point. It is notorious that in some schools jerseys are worn day after day unwashed from the beginning to the end of term. Having regard to the foulness both from within and from without with which they may become charged, it seems most important to insist upon their being frequently and thoroughly washed. Considering the compost of mud, blood, and sweat which must accumulate within their textures during the struggles of a match, it is not too much to demand that at the opening of "full dress" contests they should be absolutely clean, and that where football is the daily exercise, as at our public schools, all jerseys should be washed at least twice a week. It avails little that baths should be provided in masters' houses for ablutions after games, if next day the boys are obliged to endue their bodies with filthy clothing, to be pressed and rubbed against the open pores of their skin. In schools were the jerseys are washed twice a week, the disease is unknown, a fact which points to the real remedy for football players impetigo.

### The Factories and Workshops Bill.

The public conscience by the voice of Mr. Asquith, supported also by many voices from the other side, has led to the introduction of a Factory Bill which is all on the side of humanity. Trade may, perhaps, be restricted, profits possibly may be less, but the conditions amid which the work is done will certainly be better in those branches of industry affected by it. In many ways the Bill is a sanitary measure. Among the first matters touched is the cubic air space in factories, in regard to which it is thought desirable to set up a uniform standard for all trades. The proposal is, too, that there should be provided as a minimum 250 cubic feet per worker, and if we consider that that means a space five feet by five, or about the space of an ordinary dining-table, and ten feet high, it cannot be looked upon as excessive. It is in fact as much as is required in common lodging-houses, and less than is demanded by the Model Bye-laws for bedrooms either in lodging or tenement houses. Provision is made for enforcing a larger cubic space for each person while working overtime, and also during the time when artificial light is employed. A very important clause dealing with dangerous premises and dangerous machines has, in a way, been borrowed from the Public Health Act. As under the latter a magistrate can issue a closing order prohibiting the use of premises shown to be in a condition dangerous to health, so it is proposed that powers should be given to a Court of Summary Jurisdiction to act on the motion of an inspector, and to require that premises or machinery, reported to be dangerous or unfit for the particular process carried on, shall be disused until put into a proper condition. The provisions against sweating, or the employment of out-workers amid insanitary conditions, will no doubt be effectual for their purpose, and are calculated to put a check upon the spread of infectious disease from the dens in which a certain portion of the clothing trade is now carried on—much to the danger of the public; and the provisions against overtime being worked by young persons under eighteen will remove an evil which there is reason to believe did lifelong injury to many. The abolition of home work, "a peculiarly noxious form of overtime" in the case of children, will also be a great mercy to innumerable little ones. The extension of the provisions of the Bill to all laundries, except those of a purely domestic character, is a thing which will meet with the approval of all who have carefully inquired into the subject. It must be borne in mind, however, by hospital managers that the laundries of all public institutions will now become subject to the visits of the factory inspectors. On the whole, the Bill makes for sanitary progress by improving the conditions amid which work is done.

